# Large Acid-Evoked Currents, Mediated by ASIC1a, Accompany Differentiation in Human Dopaminergic Neurons

**DOI:** 10.3389/fncel.2021.668008

**Published:** 2021-04-27

**Authors:** Andreas Neuhof, Yuemin Tian, Anna Reska, Björn H. Falkenburger, Stefan Gründer

**Affiliations:** ^1^Department of Neurology, Institute of Physiology, RWTH Aachen University, Aachen, Germany; ^2^Department of Neurology, RWTH Aachen University, Aachen, Germany

**Keywords:** acid-sensing ion channel, neuronal differentiation, LUHMES cells, dopaminergic neuron differentiation, Ca^2+^ imaging, midbrain

## Abstract

Acid-sensing ion channels (ASICs) are proton-gated Na^+^ channels. They contribute to synaptic transmission, neuronal differentiation and neurodegeneration. ASICs have been mainly characterized in neurons from mice or rats and our knowledge of their properties in human neurons is scarce. Here, we functionally characterized ASICs in differentiating LUHMES cells, a human mesencephalic cell line with characteristics of dopaminergic neurons. We find that LUHMES cells express functional ASICs, predominantly homomeric ASIC1a. Expression starts early during differentiation with a striking surge in current amplitude at days 4–6 of differentiation, a time point where—based on published data—LUHMES cells start expressing synaptic markers. Peak ASIC expression therefore coincides with a critical period of LUHMES cell differentiation. It was associated with increased excitability, but not paralleled by an increase in ASIC1 mRNA or protein. In differentiating as well as in terminally differentiated LUHMES cells, ASIC activation by slight acidification elicited large currents, action potentials and a rise in cytosolic Ca^2+^. Applying the ASIC pore blocker diminazene during differentiation reduced the length of neurites, consistent with the hypothesis that ASICs play a critical role in LUHMES cell differentiation. In summary, our study establishes LUHMES cells as a valuable model to study the role of ASICs for neuronal differentiation and potentially also cell death in a human cell line.

## Introduction

Acid-sensing ion channels (ASICs) are proton-gated Na^+^ channels (Gründer, 2020). Among the six main ASIC subtypes, ASIC1a and ASIC2a are particularly highly expressed in the central nervous system (CNS) of rodents (Price et al., 1996; García-Añoveros et al., 1997; Lingueglia et al., 1997; Waldmann et al., 1997). ASICs are trimers and most functional channels in the rodent CNS are either ASIC1a homomers or heteromers of ASIC1a with ASIC2a or ASIC2b (Baron et al., 2002, 2008; Askwith et al., 2004; Chu et al., 2004; Wu et al., 2004; Li et al., 2010b). Because synaptic vesicles have acidic pH, ASICs contribute to synaptic transmission at excitatory synapses (Du et al., 2014; Kreple et al., 2014; Gonzalez-Inchauspe et al., 2017). Accordingly, ASIC1 is enriched in brain regions with strong excitatory input (Wemmie et al., 2003). Transient activation of ASICs, thus, modulates synaptic transmission. In addition, ASIC1 was shown to modulate the density of dendritic spines (Zha et al., 2006) and neuronal differentiation *in vitro* (O’Bryant et al., 2016). Prolonged activation of ASICs occurs in the course of ischemic stroke or inflammation and contributes to cell death in these conditions (Friese et al., 2007; Xiong et al., 2012; Wang et al., 2015; Chassagnon et al., 2017).

Most of these findings were obtained in mice or rats, and our knowledge about ASICs in the human nervous system is much more limited. In human brain, the main ASIC transcript is ASIC1a (Hoagland et al., 2010; Delaunay et al., 2012) and ASICs in human cortical neurons are predominantly homomeric ASIC1a (Li et al., 2010a). A more detailed characterization of ASICs in human neurons is clearly necessary to better understand their role in synaptic transmission, neuronal maturation and neurodegeneration in humans.

The LUHMES cell line is a subclone of the human mesenencephalic-derived cell line MESC2.10 (Lotharius et al., 2005), which was obtained by transforming committed neural precursor cells with the oncogene myc. LUHMES cells are therefore proliferating in standard medium. When myc expression is repressed by adding tetracycline, they differentiate into post-mitotic neurons (Lotharius et al., 2002). One particular advantage of LUHMES cells is the very high conversion rate (>99%) into a uniform post-mitotic population of excitable dopaminergic neurons (Scholz et al., 2011). Dopaminergic neurons of the midbrain through their projections to the striatum are essential for motor control and reward-based learning. Loss of dopaminergic neurons in the substantia nigra pars compacta (SNc) leads to Parkinson’s disease (PD). Because differentiated LUHMES cells share many characteristics with dopaminergic neurons of the SNc, they constitute a valuable model to study cellular mechanisms of neuronal differentiation, neurodegeneration (Lotharius et al., 2005) and reward-based learning.

In this study, we functionally characterized ASICs during differentiation of LUHMES cells. We found that homomeric ASIC1a is the main ASIC of LUHMES cells and found a striking transient increase of functional ASIC1a during the first week of differentiation. Activation of ASICs induced action potentials and intracellular Ca^2+^ signals and blocking ASICs reduced the length of neurites. Our study establishes LUHMES cells as a model to study ASICs in a human cell line and suggests that ASICs contribute to maturation of LUHMES cells.

## Materials and Methods

### Cell Culture

LUHMES cells were grown at 37 ^o^C in a humidified atmosphere with 5% of CO_2_. For proliferation, they were cultivated in Nunclon cell culture flasks (Thermo Fisher Scientific, Waltham, MA, USA) in Advanced DMEM/F12 medium supplemented with 2 mM L-Glu, 1× N2-supplement (Thermo Fisher Scientific, Waltham, MA, USA) and 40 μg/ml FGF, as previously described (Scholz et al., 2011). Cells were splitted 1:10 or 1:5 every 3–4 days.

To convert LUHMES cells into post-mitotic neurons, a 2-step differentiation protocol was used (Scholz et al., 2011). On day 0 (d0), the medium was exchanged by the differentiation medium: advanced DMEM/F12 medium with 2 mM L-Glu, 1 ×N2-supplement, 1 mM dibutyryl-cAMP, 1 μg/ml tetracycline and 2 ng/ml GDNF. Cells were either directly seeded in the desired density (d3 or earlier) or they were splitted on d3 and then seeded in the desired density. Depending on the experiments, cells were grown either on Nunclon cell culture dishes or on cover slips. Flasks and dishes were coated with 50 μg/μl poly-L-ornithin and 1 μg/μl fibronectin; cover slips were coated with 10 μg/μl poly-L-ornithin and 10 μg/μl laminin.

For quantification of neurite lengths, diminazene (Sigma–Aldrich; Munich, Germany) was dissolved in DMSO and added in a final concentration of 10 μM to differentiation medium; differentiation medium containing DMSO without diminazene served as a control. The same cultures from two independent differentiations were used to determine neurite outgrowth across days.

### Reverse Transcription–Quantitative Real Time PCR (qPCR)

For isolation of RNA, LUHMES cells were grown on cell culture dishes with differentiation medium or, as a control, in normal proliferation medium. On d2–d7 and additionally on d10, total RNA was isolated using RNeasy minikit (Qiagen, Venlo, The Netherlands). Concentration and quality of the RNA was measured using a NanoDrop 2000c spectrophotometer (Thermo Fisher Scientific, Waltham, MA, USA). RNAs with a 260 nm/280 nm ratio >2.00 and a 260 nm/230 nm ratio >1.80 were used for reverse transcription. First-strand cDNA was synthesized from 1 μg total RNA using QuantiTect Reverse Transcription Kit (Qiagen), yielding 20 μl cDNA. All kits were used according to manufacturer’s instructions. Contamination with genomic DNA was controlled by RT-PCR using intron-spanning primers for the reference gene glyceraldehyde-3-phosphate dehydrogenase (*GAPDH*).

For qPCR, hydrolysis probes (TaqMan probes) for *GAPDH*, *ASIC1a* and *ASIC2* were ordered from Applied Biosystems (the assay identification numbers are Hs02758991_g1, Hs00952807_m1, Hs00153756_m1). Each reaction, containing 1 μl cDNA, 1 μl TaqMan Gene Expression Assay and 5 μl of two times Rotor-Gene Probe PCR Master Mix (Qiagen), was performed in triplicates; a sample without cDNA served as negative control. qPCR was performed in a Rotor-Gene Q (Qiagen), starting with a long denaturation phase (10 min, 95°C), followed by 40 cycles with denaturation (15 s, 95°C) and annealing/elongation (60 s, 60°C/72°C). Experiments were repeated with RNA from two independent cell batches and analyzed using the ΔCt method. Efficiency of each probe was determined by a standard curve and was close to 100%. Results are reported as relative levels of *ASIC*/*GAPDH* mRNA.

### Immunoblotting

LUHMES cells were lysed with RIPA buffer (25 mM Tris-Cl pH 7.6, 0.1% SDS, 150 mM NaCl, 1% Triton-X-100, 1% sodium deoxycholate, 1% PMSF, and 1% proteinase inhibitor cocktail; Roche). Proteins were quantified using a kit (Micro BCA; Thermo Fisher Scientific, Waltham, MA, USA) and the same amount of protein was separated by SDS-PAGE (10%). Proteins were transferred to PVDF membranes (Roche, Mannheim, Germany), and probed overnight at 4°C with the following primary antibodies: mouse monoclonal anti-ASIC1 (1:1,000 dilution, NeuroMab #75-277) and mouse monoclonal anti-acetylated tubulin (1:5,000 dilution, Sigma–Aldrich #T7451). The anti-ASIC1 antibody was derived against a fusion protein from the cytoplasmic C-terminus of mouse ASIC1; 65 of the 67 amino acids of this fusion protein are identical in human ASIC1. It was validated by NeuroMab in immunoblots from ASIC1 knock-out tissue. Blots were visualized using secondary HRP-conjugated anti-rabbit (Invitrogen #UB280570) or anti-mouse antibodies (1:10,000 dilution, Invitrogen #2122350) and Super Signal West Pico Chemiluminescent Substrate (Thermo Fisher Scientific, Waltham, MA, USA). Bands of the expected size were quantified using ImageJ, and density for ASIC1a was normalized to tubulin.

### Electrophysiology

Electrodes with a resistance of 5–10 MΩ were pulled from borosilicate glass with a DMZ-Universal Puller (Zeitz Instruments GmbH, Martinsried, Germany). Electrodes were filled with an intracellular solution containing 10 mM NaCl, 10 mM KCl, 25 mM HEPES, 70 mM K-Gluconate, 10 mM EGTA and 1 mM MgCl_2_; pH was adjusted to 7.25 with tetramethylammonium hydroxide (TMAOH) solution. The extracellular solution in the bath chamber contained 100 mM NaCl, 5.4 mM KCl, 10 mM HEPES or MES, 2 mM CaCl_2_ and 1 mM MgCl_2_; pH was adjusted with TMAOH. Whole cell currents were recorded using a patch-clamp amplifier (Axopatch 200B), the Axon-CNS (Digidata 1440A) and Clampex software (Molecular Devices). Data were filtered at 1 kHz with low-pass filter, and stored continuously on a computer hard disc and analyzed using pCLAMP software. For voltage clamp, the membrane voltage was clamped to −70 mV, and the sampling rate was 4 kHz. PcTx1 was purchased from Smartox biotechnology. For current clamp, the membrane current was clamped to 0 pA for the gap free protocol. For measurements of the rheobase, ten long (1 s) depolarizing current pulses (increments of 1, 2, 5, or 10 pA) were delivered; data was sampled at a rate of 20 kHz.

### Ca^2+^ Imaging

For cell fluorescence measurements, LUHMES cells grown on glass coverslips were mounted in a cell chamber and perfused with bath solution at room temperature. Fluorescence was measured continuously on an inverted microscope (IX71, Olympus, Chromaphor) using a Fluar 20×/0.75 objective (Olympus) and Till Vision real-time imaging software (Till Photonics). Cells were loaded for 15 min at 37°C with 2 μM Fura-2-AM (Molecular Probes) in bath solution. Fura-2 was excited at 340/380 nm, and the emission was recorded between 470 and 550 nm using a sensicam CCD camera (PCO imaging). Acquisition and data analysis were done using Till Vision software. Amiloride and nimodipine were purchased from Sigma–Aldrich.

### Data Analysis

To analyze the expression of functional ASICs, the peak current elicited by pH 6.0 was divided by the electrical capacity to obtain the current density. The time constant τ_des_ was determined by fitting a single exponential function to the current decay using the software Igor Pro (WaveMetrics, Tigard, OR, USA). The concentration-response curve in Figure 3 was obtained by a fit to a Hill function:

**Figure 1:**
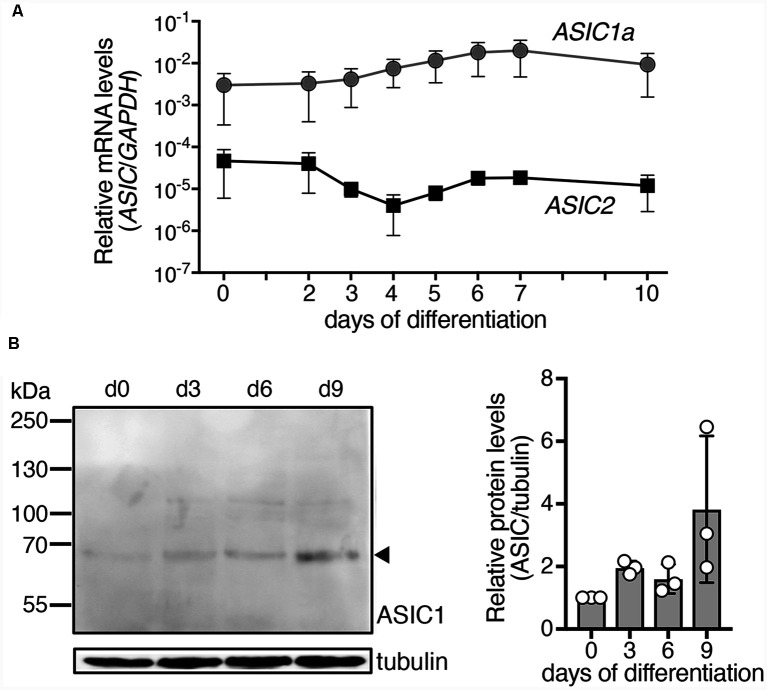
Expression of *ASIC1a* and *ASIC2* mRNAs and ASIC1a protein during differentiation of LUHMES cells into post-mitotic neurons. **(A)** Expression of ASIC mRNA was quantified using quantitative real time PCR and was normalized to the housekeeping gene glyceraldehyde 3-phosphate dehydrogenase (*GAPDH*), *n* = 2. Note that errors bars have different length above and below of the data points, because of the logarithmic scale of the y axis. **(B)** Left, representative Western blot showing expression of ASIC1 protein in LUHMES cells; tubulin was used as control. Right, summary of three Western blots; expression was normalized to tubulin and to d0.

**Figure 2:**
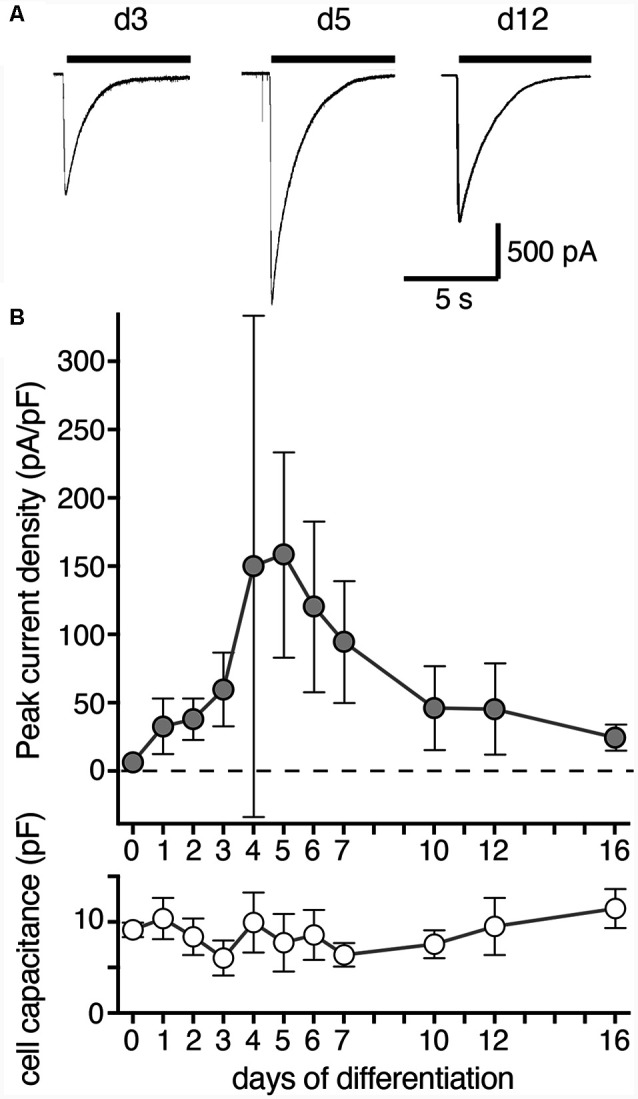
Density of ASIC peak currents during differentiation of LUHMES cells into post-mitotic neurons. **(A)** Representative current traces from d3, d5 and d12. ASIC currents were elicited by a drop in pH from 7.4 to 6.0 and cells analyzed by whole cell patch-clamp. **(B)** Summary of current density and cell capacitance for different days of differentiation as indicated (mean ± SD; *n* = 4–12 from two independent experiments). Note that the current density on d4 was highly variable and not normally distributed with some cells having a small density like on d3 and others a large density like on d5. Current amplitude ranged from 36 to 3,603 pA for individual cells on different days of differentiation.

**Figure 3:**
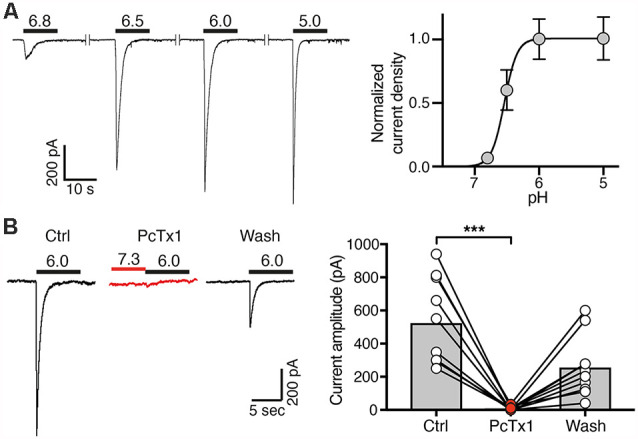
Concentration-response curve and PcTx1 sensitivity of ASICs in post-mitotic LUHMES cells. **(A)** Cells were cultivated in differentiation medium for 6 days and were then analyzed by whole cell patch-clamp in voltage clamp mode. Left, representative ASIC currents elicited by transient pH changes from pH 7.4 to values between 6.8 and 5.0 for 10 s. For recovery of ASICs, cells were washed with a solution of pH 7.4 for 30 s. Right, concentration response curve. Currents were normalized to the current at pH 5.0, which had a density of 120 pA/pF. Line represents a fit to the Hill function. *n* = 11 cells from two independent experiments. **(B)** PcTx1 sensitivity of ASICs on d4–d8. Left, representative whole cell current traces, each elicited by a 10 s-pulse of pH 6 from conditioning pH 7.3. Traces were acquired before, during and after application of 30 nM PcTx1 for 2 min. Right, summary of peak current amplitude before, during and after PcTx1 application. *n* = 10. ***p = 0.0001 (paired *t*-test).

$$I=\frac{1}{\left(1+{\left(\frac{\left[H^{+}\right]}{EC_{50}}\right)}^{H}\right)}$$

where *I* is the current, [*H*^+^] is the proton concentration, *EC*_50_ is the concentration at which 50% of the maximal current is obtained, and *H* is the Hill coefficient (GraphPad Prism).

Ca^2+^ signals elicited by acidic pH were quantified by subtracting the baseline values at 340/380 nm before application of acidic pH from the peak values during application of acidic pH. For application of inhibitors (nimodipine or amiloride, or no additional treatment as control), Ca^2+^ signals during application of the inhibitor were normalized to the Ca^2+^ signals before application of the inhibitor (3rd/2nd peak).

Data are reported as mean ± SD, except for the data in Figure 3A, which are reported as mean ± SEM, to indicate the precision of the data points used to draw the graph. Statistical analyses were conducted with Prism 7.0 (GraphPad Software, San Diego, CA, USA). Normal distribution of each dataset was tested using the D’Agostino–Pearson omnibus K2 test. For data, which was normally distributed according to the omnibus K2 test, we used two-tailed paired or unpaired Student’s *t*-test, as appropriate, when comparing two groups and a one-way or two-way analysis of variance (ANOVA), followed by Tukey’s multiple comparison test, when comparing more than two groups. For data, which was not normally distributed according to the omnibus K2 test, we either used a Mann–Whitney test (two groups) or a Kruskal–Wallis test followed by Dunn’s multiple comparison test (more than two groups). Data, which were categorized in two classes (AP vs. no AP), was analyzed using Fisher’s exact text. *p* ≤ 0.05 was considered as significant.

The total length of neurites of single cells was determined on d1–d5 using the plugin IC Neuron of the application IC Capture (The Imaging Source, Bremen, Germany).

## Results

### *ASIC1a* mRNA Is the Predominant ASIC Transcript in LUHMES Cells

The *ASIC1* gene codes for ASIC1a and ASIC1b and *ASIC2* for ASIC2a and ASIC2b, respectively. Expression levels of *ASIC1* variant a (*ASIC1a*) and *ASIC2* were determined during differentiation of LUHMES cells by quantitative real time PCR (qPCR; Figure 1A). For *ASIC2*, we used primers that detect mRNAs for variant a (ASIC2a) and for variant b (ASIC2b). Nevertheless, at any time point, expression of *ASIC2* was much lower (60- to >1,000-fold) than that of *ASIC1a*, indicating that *ASIC1a* mRNA was the primary ASIC transcript in these cells. The average expression of *ASIC1a* rose by <10-fold during differentiation of LUHMES cells. Expression of ASIC1a protein in LUHMES cells was confirmed by Western blot analysis (Figure 1B). ASIC1a abundance was about 2-fold higher after 3 days of differentiation (d3) compared with undifferentiated LUHMES cells (d0), but there was no further increase with longer time in differentiation medium and the difference was not statistically significant (*p* = 0.09, one-way ANOVA).

### ASIC Current Amplitude Peaks on Day 6 of LUHMES Cell Differentiation Into Neurons

We then determined the expression of functional ASICs in LUHMES cells during differentiation using the whole cell patch clamp technique. We activated ASICs by a pH drop from 7.4 to 6.0. In proliferating LUHMES cells on d0, which have already a definite neuronal commitment (Scholz et al., 2011), ASIC current density was very small (<10 pA/pF; *n* = 3). Peak current density strongly increased from d3 to d4 and d5 (increase from 59.6 pA/pF on d3 to 158.6 pA/pF on d5; *p* < 0.001; *t*-test; Figure 2). During the next 5 days, peak current density decreased again to its initial levels. Thus, there was a striking, transient surge in ASIC peak current density during the first 5 days of differentiation, which was only partially mirrored by the mRNA and protein expression levels (Figure 1). ASIC currents had a large amplitude, with a mean amplitude of 1,084 ± 479 pA (mean ± SD) on d5.

### Homomeric ASIC1a Is the Predominant Functional ASIC in LUHMES Cells

Next, we determined the ASIC current amplitude at d4–d7 at different pH values, revealing half-maximal activation at pH 6.6 ± 0.1 on d4 (mean ± SD; *n* = 12), at pH 6.5 ± 0.2 on d5 (*n* = 11), at pH 6.5 ± 0.1 on d6 (*n* = 11) and at pH 6.6 ± 0.1 on d7 (*n* = 12) and saturating amplitudes at pH 6.0 (Figure 3A). This result strongly suggests that the highly proton-sensitive homomeric ASIC1a mediates the surge in ASIC current. Nearly complete inhibition of ASIC currents by PcTx1 (30 nM) at d4–d8 confirmed the presence of mainly homomeric ASIC1a in differentiating LUHMES cells (Figure 3B). Heteromeric ASIC1a/2b has similar functional properties as homomeric ASIC1a, including sensitivity to protons and to PcTx1 (Sherwood et al., 2011). Therefore, the presence of functional ASIC1a/2b in LUHMES cells cannot be excluded. Low expression of the *ASIC2* gene (Figure 1A), however, suggests low abundance of ASIC2b in LUHMES cells. In contrast, the presence of ASIC1a homomers in differentiating LUHMES cells would be consistent with the mRNA analysis (Figure 1A).

**Figure 4:**
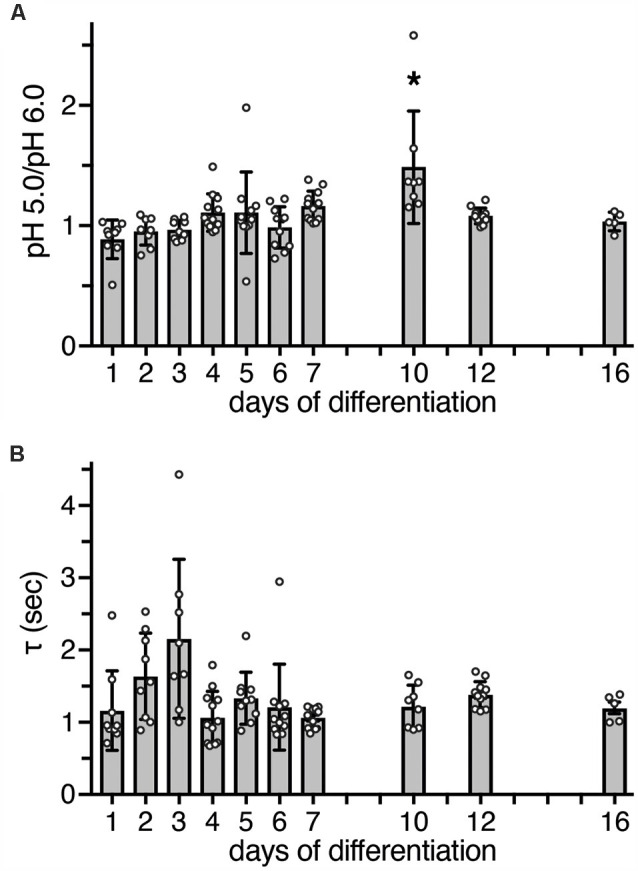
Functional characteristics of ASIC currents *between* d1 *and* d16. **(A)** Bars represent the peak amplitude elicited by pH 5.0 divided by the peak amplitude elicited by pH 6.0. Currents were elicited by a pH drop from 7.4 to 6.0 or 5.0, respectively, for 10 s. The ratio at d10 was significantly different from d1 (*p* < 0.0001), d2 (*p* = 0.001), d3 (*p* = 0.001), and d6 (*p* = 0.011, Kruskal-Wallis test followed by Dunn’s test). *p < 0.05. **(B)** Bars represent τ_des_ of ASIC currents at different days of differentiation; *p* > 0.05 on all days (Kruskal-Wallis test followed by Dunn’s test). *n* = 5–12 from two independent experiments.

To determine whether heteromeric ASIC1a/2a is also present during differentiation, we measured peak currents elicited by pH 6.0 and by pH 5.0. While homomeric ASIC1a is already maximally activated by pH 6.0 or by slightly more acidic pH (Waldmann et al., 1997; Babini et al., 2002), current amplitudes of heteromeric ASIC1a/2a only saturate around pH 5.0 or at slightly more acidic pH (Bassilana et al., 1997; Bartoi et al., 2014; Joeres et al., 2016). Thus, a ratio of *I*_pH5.0_/*I*_pH6.0_ close to 1 is indicative of homomeric ASIC1a; the presence of ASIC2a should increase this value up to 2 for a pure heteromeric channel. In LUHMES cells, the *I*_pH5.0_/*I*_pH6.0_ was close to 1 on almost all days; only on d10 it was 1.5 (*p* < 0.05 compared to d1, d2, d3 and d6; Kruskal–Wallis test; Figure 4A).

As a further characteristic that differentiates between different ASIC subtypes, we determined the desensitization time constant τ_des_ after activation with pH 6.0. At pH 6.0, human ASIC1a desensitizes with a time constant τ_des_ of 1–2 s (Xu et al., 2018; Vaithia et al., 2019). To our knowledge, the time constant of heteromeric human ASIC1a/2a has not yet been reported, but ASIC1a/2a from rodents desensitizes with a τ_des_ <1 s (Benson et al., 2002; Hattori et al., 2009). A time constant τ_des_ of ASIC currents in LUHMES cells could be well fitted with a single exponential function, suggesting a predominantly homogenous population of ASIC channels. Moreover, on all days, τ_des_ was similar and >1 s (*p* > 0.05, Kruskal-Wallis test; Figure 4B). Thus, although on d10 the ratio of *I*_pH5.0_/*I*_pH6.0_ was 1.5, the presence of a heteromeric ASIC1a/2a on d10 was not confirmed by the analysis of τ_des_. Therefore, these results, collectively, suggest that ASIC currents were carried mainly by homomeric ASIC1a and perhaps ASIC1a/2b during the entire period of differentiation.

### Activation of ASICs by Slight Acidification Elicits Action Potentials in LUHMES Cells

**Figure 5:**
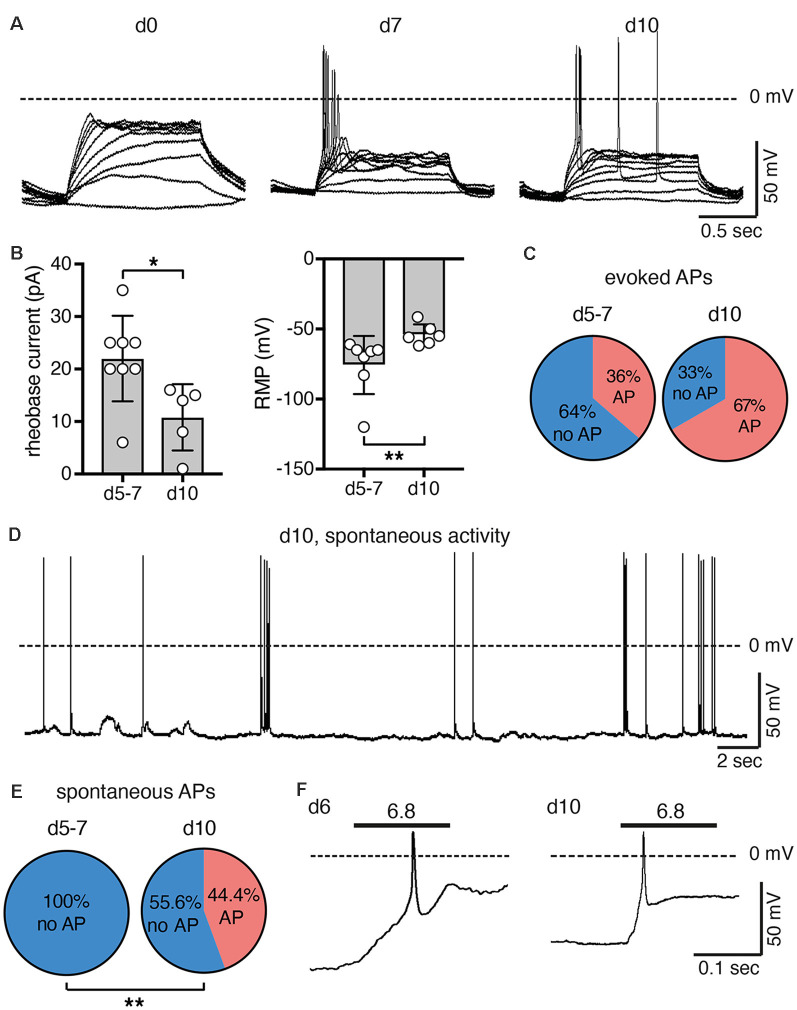
Excitability of LUHMES cells during differentiation. **(A)** Representative current clamp recordings from LUHMES cells on d0, d7 and d10. Depolarizing step current pulses elicited APs only in differentiated LUHMES cells. **(B)** Summary of the rheobase and the resting membrane potential (RMP) at different times of differentiation (*n* = 5–8); *p < 0.05, **p < 0.01 (Mann-Whitney test). **(C)** Pie chart illustrating the proportion of cells, which responded with APs after pulse stimulation, at d5–d7 (8 out of 22 cells) and at d10 (six out of nine cells). **(D)** Example of spontaneous APs from LUHMES cells at d10. The trace is representative for four out of nine cells. **(E)** Pie chart illustrating the proportion of cells, which had spontaneous APs at d5–d7 and at d10. At d10, significantly more cells had spontaneous APs (*p* = 0.004, Fisher‘s exact test). **(F)** Examples of APs elicited by steps to pH 6.8 (*n* = 2 for each day).

We next assessed excitability of undifferentiated LUHMES cells (d0) and of cells on d5–d7 or on d10. We stimulated the cells by applying 1 s-current pulses of increasing amplitude and monitored the membrane potential in current clamp mode. In undifferentiated cells (d0), even depolarizing close to 0 mV did not elicit an action potential (AP). In contrast, APs were readily elicited at d5 or later (Figure 5A). With longer time of differentiation, smaller current pulses were needed to reach threshold and elicit APs. The rheobase current decreased 2-fold from d5/d7 to d10 (*p* = 0.02; Mann–Whitney test), which can be explained by the less negative resting membrane potential (RMP) after longer differentiation (*p* = 0.002; Mann–Whitney test; Figure 5B). The proportion of cells, in which APs could be elicited, almost doubled from d5/d7 to d10 (*p* = 0.23, Fisher‘s exact test; Figure 5C). Spontaneous APs where not observed in any of the 22 cells on d5/d7, but we observed spontaneous APs in four out of nine cells on d10 (*p* = 0.004, Fisher‘s exact test), some of which occurred in bursts (Figures 5D,E). Taken together, although we did not systematically characterize excitability over the whole period of differentiation, these results suggest that excitability of LUHMES cells gradually increased during differentiation. This interpretation is in line with a previous study, which reported that current density of voltage-gated Na^+^ channels gradually increases from d3 to d11, that approximately 40% of cells were spontaneously active up to d9, and that all cells generated spontaneous APs on d10–d12 (Scholz et al., 2011).

After examining general excitability, we next tested whether activation of ASICs was sufficient to reach threshold and elicit APs. Modest acidification to pH 6.8 indeed depolarized the membrane potential and elicited APs at both d6 and d10 (Figure 5F). The larger rheobase on d6 (Figure 5B) may be compensated for by the larger ASIC current density at this stage (Figure 2).

### ASICs Are Involved in Neurite Outgrowth During LUHMES Cell Differentiation

**Figure 6:**
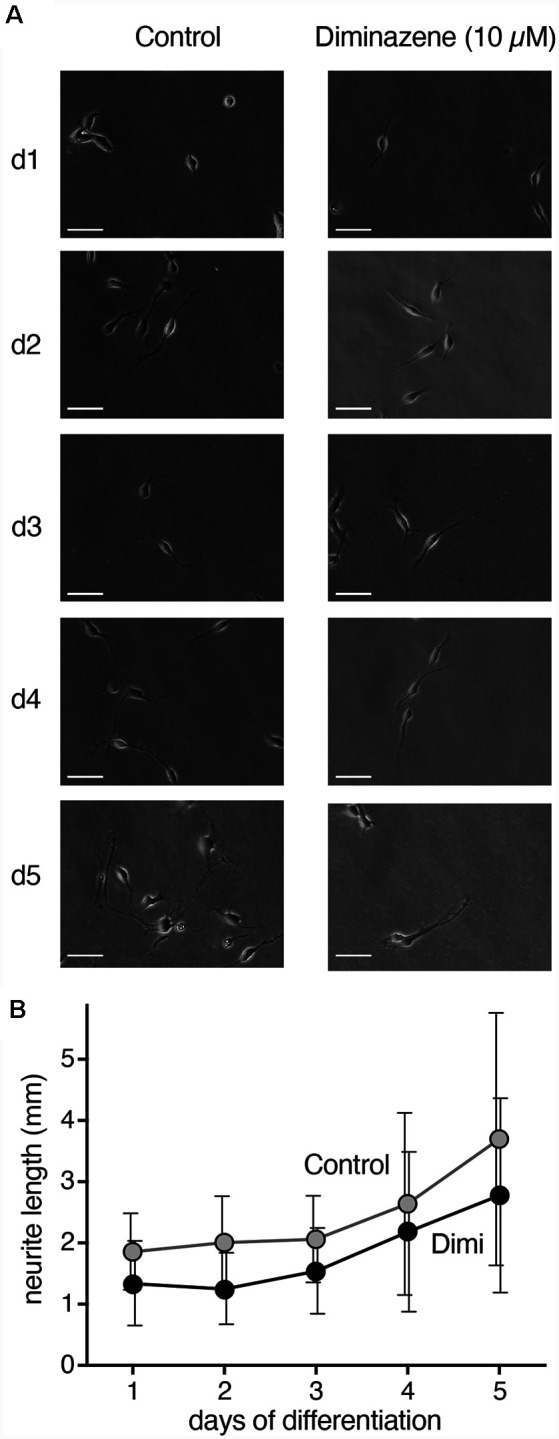
Neurite length during differentiation of proliferating LUHMES cells into post-mitotic neurons. **(A)** Representative images of LUHMES cells at d1–d5, with and without incubation in diminazene (10 μM). **(B)** Quantification of the total length of neurites of single cells at d1–d5. Dimi, diminazene. *n* = 26–60 neurites from 12–24 different cells.

The transient surge in ASIC current density during differentiation of LUHMES cells suggest their involvement in the differentiation process. Indeed, blocking ASICs was reported to reduce differentiation of NS20Y cells, a murine neuroblastoma-derived cell line (O’Bryant et al., 2016). To determine whether ASICs are involved in the differentiation of LUHMES cells, we applied the isoform-unselective ASIC pore blocker (Schmidt et al., 2017) diminazene (10 μm) during the first 5 days of differentiation and assessed the length of neurites as a measure for neuronal differentiation. In control cells, the length of neurites almost doubled from d1 to d5 (Figure 6). Neurite lengths of diminazene-treated cells were overall shorter than for control cells on all days investigated (Figure 6; *p* < 0.001, two-way ANOVA). Diminazene has, to our knowledge, not previously been tested on human ASICs, but the amino acids in the outer ion pore that are crucial for inhibition (Schmidt et al., 2017) are completely conserved in human ASICs, arguing that diminazene will also inhibit human ASICs. Although we cannot exclude that diminazene might also have other targets, this result supports the hypothesis that ASICs play an important role in neuronal development.

### ASIC Activation Elicits Ca^2+^ Signals in Differentiated LUHMES Cells

**Figure 7:**
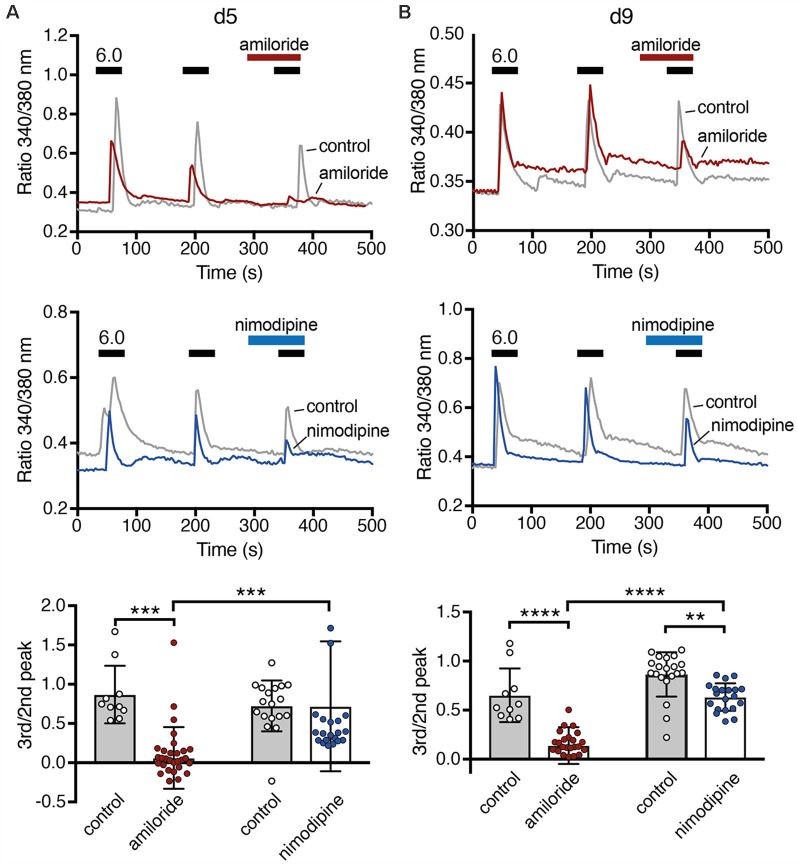
Ca^2+^ responses of LUHMES cells after acidic stimulation. **(A)** Top, representative recordings of intracellular Ca^2+^ by Fura-2 in LUHMES cells at d5. Cells were repeatedly stimulated with pH 6 (black bars). For some cells, 100 μM amiloride was pre- and co-applied during the third stimulation (red bar). The gray trace represents the mean response of >10 control cells from one coverslip and the red trace of >10 cells from another coverslip exposed to amiloride. Middle, as for the top panel but with 10 μM nimodipine instead of amiloride. The blue trace is from cells exposed to nimodipine. Bottom, summary data. **(B)** As in **(A)** but for d9, **p < 0.01, ***p < 0.001, ****p < 0.0001 (two-way ANOVA).

A rise in cytosolic Ca^2+^ is one of the main pathways by which excitation and excitability affect differentiation, morphology and degeneration. To determine whether ASICs could modulate differentiation of LUHMES cells through intracellular Ca^2+^, we tested whether ASIC activation can induce a Ca^2+^ response in LUHMES cells. On d5 and d9, stimulation with pH 6.0 evoked a robust, transient Ca^2+^ signal (Figure 7), which would be expected if it was mediated by ASIC activation. The response gradually decreased upon repeated stimulation, which can be explained by tachyphylaxis of ASIC1a (Chen and Gründer, 2007). On both days, the isoform-unselective ASIC blocker amiloride almost completely inhibited the Ca^2+^ response (*p* < 0.001, two-way ANOVA; Figure 7). Although amiloride has other targets like Na^+^/H^+^ exchangers, this result suggests that the Ca^2+^ influx in LUHMES cells was indeed ASIC-dependent. To test whether the Ca^2+^ influx was through ASIC itself, or through activation of voltage-gated Ca^2+^ channels (Ca_V_), we applied the L-type Ca_V_ blocker nimodipine. Nimodipine significantly reduced the Ca^2+^ influx induced by pH 6.0 on d9 (*p* = 0.003) but not on d5 (Figure 7). At d5, there were two outliers with unusually large Ca^2+^ signals (Figure 7A). Excluding them would reveal significantly reduced Ca^2+^ influx by nimodipine also at d5 (*p* = 0.014). On both d5 and d9, nimodipine reduced Ca^2+^signals less strongly than amiloride (*p* = 0.002 at d5, *p* = 0.0002 when the outliers were removed; *p* = 0.006 at d9; *p-values* for the interaction *between* amiloride *and* nimodipine). Thus, although acidic pH-induced Ca^2+^ signals were at least on d9 partly through indirect activation of voltage-gated Ca^2+^ channels, the remaining Ca^2+^might have entered the cell directly *via* ASIC1a.

## Discussion

Our results reveal expression of functional ASICs in human dopaminergic LUHMES cells, consistent with the previous description of ASICs in dopaminergic neurons of mouse midbrain slices (Pidoplichko and Dani, 2006), isolated neurons of the mouse substantia nigra pars compacta (SNc; Arias et al., 2008) and the human cell line SH-SY5Y, which is derived from a bone marrow biopsy of a patient with neuroblastoma, expresses dopaminergic markers and releases catecholamines (Xiong et al., 2012). Moreover, our results consistently suggest that homomeric ASIC1a is the main ASIC during the entire differentiation of LUHMES cells into postmitotic cells with neuronal characteristics; some contribution by heteromeric ASIC1a/2b can also not be excluded. First, qPCR revealed *a* >60-fold higher abundance of ASIC1a mRNA than ASIC2a and ASIC2b mRNA combined (Figure 1A). Second, apparent *EC*_50_ of this ASIC was pH 6.5 (Figures 3A, 4A), indicative of highly proton-sensitive homomeric ASIC1a or ASIC3. Third, PcTx1, which at pH 7.4 specifically inhibits homomeric ASIC1a (Escoubas et al., 2000; Joeres et al., 2016), inhibited LUHMES cell ASICs almost completely (Figure 3B). Finally, τ_des_ was >1 s (Figure 4B), excluding heteromeric ASIC1a/2a as well as homomeric ASIC3. This is consistent with the characteristics of ASICs in mouse midbrain slices (Pidoplichko and Dani, 2006)—even though the apparent *EC*_50_ in these measurements was pH 5.5; homomeric ASIC1a was also shown to at least contribute to the currents in SH-SY5Y cells (Xiong et al., 2012).

LUHMES cells are human, dopaminergic cells that show neuron-like electrical properties and morphology (Lotharius et al., 2002; Scholz et al., 2011). They are derived from immortalized embryonic midbrain and show a very high conversion rate into dopaminergic neurons, which sets them apart from cells derived from human induced pluripotent stem cells (iPSC) where the rates of neuronal and dopaminergic phenotypes are typically much lower (Xi et al., 2012; Doi et al., 2014). Still, the phenotype of neurons is not only shaped by their origin, but also by their environment, and this environment is certainly different in the brain than in a culture dish. Furthermore, the human midbrain comprises at least two populations of dopaminergic neurons that are differentially affected in Parkinson’s disease (Gibb and Lees, 1991; Dauer and Przedborski, 2003; Halliday et al., 2014). To our knowledge, LUHMES cells are the culture model that best model the population of dopaminergic SNc neurons that degenerate in PD, but they are still a model. Our conclusion that human midbrain dopaminergic neurons express functional ASICs, mainly homomeric ASIC1a, therefore needs to be confirmed by PCR and immunoblots from human mesencephalon.

Dopaminergic neurons of the midbrain receive excitatory input from the cortex and, because ASICs contribute to the postsynaptic current at excitatory synapses (Du et al., 2014; Kreple et al., 2014; Gonzalez-Inchauspe et al., 2017), it is conceivable that ASIC1a contributes to excitation of midbrain dopaminergic neurons. Accordingly, ASIC currents had a large amplitude in LUHMES cells (Figure 2) and activation of ASICs could elicit APs (Figure 5) and intracellular Ca^2+^ signals (Figure 6) in differentiated LUHMES cells. Similarly, ASICs were shown to contribute to catecholamine secretion in SH-SY5Y cells (Xiong et al., 2012), a process dependent on intracellular Ca^2+^. These findings therefore suggest that ASIC1a may modulate excitability, Ca^2+^ homeostasis and dopamine secretion in human dopaminergic neurons.

Midbrain dopaminergic neurons are spontaneously active pacemakers. Activity patterns and Ca^2+^ homeostasis are important factors that contribute to their degeneration in Parkinson’s disease (PD; Duda et al., 2016). Understanding the contribution of ASIC1a for the excitability and Ca^2+^ homeostasis in human dopaminergic neurons will therefore provide insight into the pathogenesis of PD and potentially provide targets for new treatment strategies. Neurodegeneration in PD likely , results not from a single insult, but from a network of events including in addition mitochondrial impairment, protein misfolding and inflammation (Lang and Espay, 2018). Sensing protons secreted by the inflammatory response might therefore constitute another role of ASIC1a in PD pathogenesis. Dopaminergic neurons of the SNc project to the striatum, where they modulate direct and indirect pathways through excitatory D_1_ receptors and inhibitory D_2_ receptors, respectively. This modulation is important for movement control; the loss of dopamine secretion in the striatum causes PD motor symptoms. In order to overcome current impediments of medical therapy in PD, non-dopaminergic pathways to modulate dopamine secretion and striatal physiology are increasingly investigated (Zhai et al., 2019). The contribution of ASIC1a could be studied in LUHMES cells and reveal new insights into movement control by midbrain dopaminergic neurons and potentially offer new treatment strategies for PD.

Dopaminergic neurons also project from the midbrain to the nucleus accumbens (NAc), which is homologous to the motor striatum and is involved in reward-based learning. ASIC1a is abundantly expressed in the NAc (Wemmie et al., 2003) and contributes to synaptic transmission and affects cocaine-conditioned learning (Kreple et al., 2014). ASIC1 is upregulated in the NAc upon either chronic systemic injection of cocaine (Zhang et al., 2009) or of amphetamines (Suman et al., 2010), suggesting that ASIC could be involved in drug addiction and reward-based learning. Although the NAc seems to have a key role for the modulation of drug-based learning by ASIC1a (Kreple et al., 2014), expression of ASIC1a in the SNc suggests that it might contribute to reward-based learning also *via* modulation of dopamine-release. This possibility warrants further investigation.

Our study revealed a striking transient surge in ASIC current density at d4–d6 of differentiation of LUHMES cells, when current densities were 3- to 4-fold higher than at d2 or d12 and reached 160 pA/pF (Figure 2). For comparison, the average ASIC current density was 30 pA/pF in a study using human cortical neurons (Li et al., 2010a) and 22 pA/pF in sensory neurons derived from human pluripotent stem cells (Young et al., 2014). ASIC current amplitude in human glioblastoma derived stem cell lines was variable but mostly <300 pA, corresponding to <15 pA/pF (with a mean cell capacitance of 20 pF; Tian et al., 2017). Thus, while the ASIC current density up to d2 and after d10 was comparable to other human neurons, at d5 it was substantially higher. This surge was not paralleled or preceded by a similar increase in ASIC1a mRNA of protein (Figure 1), suggesting that the increase in functional ASIC1a on the surface of LUHMES cells was due either to a more efficient forward trafficking of preformed channels that were for example residing in the ER or to a reduced endocytosis of functional channels. It has previously been shown that in CHO cells and mouse cortical neurons, most of the ASIC1a pool resides in intracellular compartments, mainly the ER, and that the ASIC current density in these cells can be rapidly (within 1 h) increased upon insulin depletion (Chai et al., 2010). The surge in ASIC current density during differentiation of LUHMES cells cannot be attributed to the depletion of insulin, however, because the N2 supplement in proliferation and in differentiation medium contains insulin. Moreover, the increase in ASIC current amplitude in our study occurred on the order of days while in the previous study after insulin depletion it occurred on the order of hours (Chai et al., 2010). Although the mechanism for the strong surge in ASIC current density during the first week of differentiation remains unknown, a more efficient forward trafficking is an attractive hypothesis.

The surge in ASIC current density parallels the appearance of excitability during differentiation of LUHMES cells (Figure 5). A previous study also found an almost linear increase in current density of voltage-gated Na^+^ channels from d3 to d11 (Scholz et al., 2011). The high ASIC current density might ensure a robust depolarization by acidic stimuli when LUHMES cells are not terminally differentiated. As activation of ASIC1a at d5 also induced robust Ca^2+^ signals (Figure 6), ASICs might be essential for maturation of LUHMES cells. In support of this idea, the surge in ASIC expression coincides with a strong increase of synaptic markers in LUHMES cells, which occurs during the first day and peaks on d6 (Scholz et al., 2011). Moreover, a study characterizing ionic conductance’s in rat neuronal precursor cells reported that amiloride-sensitive proton-activated Na^+^ currents were of large amplitude and appeared in parallel to voltage-gated Na^+^ and Ca^2+^ channels but preceded glutamate-gated ion channels by several days (Grantyn et al., 1989). The authors suggested that the proton-activated Na^+^ currents are present during the earliest stages of neuronal development and may regulate a variety of Ca^2+^-catalyzed processes during development of neurons (Grantyn et al., 1989). Our own results support this hypothesis. After d6, when LUHMES cells became more easily excitable and the resting membrane potential less negative with a decrease in rheobase current (Figure 5), ASIC1a current density decreased again (Figure 2).

It has previously been shown that transfection of ASIC1a into hippocampal slices increased the density of dendritic spines (Zha et al., 2006). Decreasing ASIC1a protein levels by siRNA had the opposite effect (Zha et al., 2006). In contrast, ASIC1a-deficient mice had no difference in hippocampal spine density compared with wildtype mice, suggesting compensatory mechanisms during development of ASIC1-knockout mice (Zha et al., 2006). The effects of ASIC1 on spine density required CaMKII, suggesting an important role for acid-induced Ca^2+^ increases (Zha et al., 2006). Our results show that in the presence of the ASIC pore blocker diminazene (Schmidt et al., 2017), the length of neurites was decreased (Figure 7), suggesting a role for ASIC1a for the maturation of LUHMES cells into neurons. Although preliminary, these results are similar to previous findings in mouse neuroblastoma-derived NS20Y cells (O’Bryant et al., 2016). Thus, one specific role for ASIC1a during neuronal maturation might be the Ca^2+^-dependent maturation of neurites and dendrites.

In summary, our study establishes dopaminergic LUHMES cells as a valuable model to study the role of ASICs during neuronal development in a human cell line.

## Data Availability Statement

The original contributions presented in the study are included in the article, further inquiries can be directed to the corresponding author/s.

## Author Contributions

AN performed the qPCR, most voltage clamp experiments and determined neurite lengths. YT performed the Western blots, current clamp experiments and Ca^2+^ imaging. AR performed voltage clamp experiments with PcTx1. AN and YT analyzed the data and participated in the design of the study. SG and BF conceived the study, designed and coordinated the study, and participated in data analysis. AN, SG, and BF drafted the manuscript. All authors contributed to the article and approved the submitted version.

## Conflict of Interest

The authors declare that the research was conducted in the absence of any commercial or financial relationships that could be construed as a potential conflict of interest.

## Abbreviations

ASIC: acid-sensing ion channel
CNS: central nervous system
LUHMES: Lund human mesencephalic
GAPDH: glyceraldehyde 3-phosphate dehydrogenase
mRNA: messenger RNA
qPCR: quantitative polymerase chain reaction
PcTx1: psalmotoxin 1
AP: action potential
Ca_v_: voltage-gated Ca^2+^ channel
SNc: substantia nigra pars compacta
PD: Parkinson’s disease.
